# The effect of fully threaded cannulated screws in sliding fixation of femoral neck fractures: a retrospective study

**DOI:** 10.1186/s13018-025-06611-4

**Published:** 2026-01-10

**Authors:** Mingwang Jia, Chenning Ding, Jiahui Zhang, Xing Han, Xin Zhao, Xiguang Sang

**Affiliations:** 1 Department of Emergency Surgery and Orthopaedic Surgery, Qilu Hospital of Shandong University, Jinan, China; 2 Cheeloo College of Medicine, Shandong University, Jinan, China

## Abstract

**Background:**

The purpose of this study was to compare the clinical efficacy of fully-threaded cannulated screws (FCS) and partially-threaded cannulated screws (PCS) in femoral neck fractures (FNFs).

**Methods:**

This retrospective study included 141 patients with FNFs treated with cannulated screw internal fixation between January 2019 and December 2023. Based on the implant type, patients were allocated into two groups: the FCS group (n = 61) and the PCS group (n = 80). Baseline demographic and clinical characteristics were collected and compared between the two groups, including age, sex, body mass index (BMI), comorbidities and fracture classification. The primary outcome measure was the incidence of postoperative complications at final follow-up, including femoral neck shortening, osteonecrosis of the femoral head (ONFH), nonunion, and internal fixation failure. The secondary outcomes included the following measures: the Garden’s alignment index and surgical data.

**Results:**

A total of 141 patients with FNF were enrolled in this study, comprising 80 in the PCS group and 61 in the FCS group. The cohort had a mean age of 44.18 ± 12.5 years, with 87 (61.7%) males and 54 (38.3%) females. Based on preoperative imaging, 84 (59.6%) of the fractures were classified as displaced. The FCS group demonstrated significantly lower rates of both ONFH (8.2% vs 21.3%, *p* = 0.034) and femoral neck shortening (8.2% vs 25%, *p* = 0.010) compared with the PCS group. No statistically significant differences were observed in nonunion (4.9% vs 6.3%, *p* = 0.735) or internal fixation failure rates (0% vs 3.8%, *p* = 0.126). Subgroup analysis demonstrated significantly higher rates of ONFH (22.6% vs 5.3%, *p* = 0.005) and femoral neck shortening (26.2% vs 5.3%, *p* = 0.001) in displaced (Garden III–IV) fractures compared with non-displaced fractures, as well as elevated complication rates in Pauwels III fractures versus Pauwels I–II fractures (ONFH: 19.8% vs 5.0%, *p* = 0.029; femoral neck shortening: 21.8% vs 7.5%, *p* = 0.045).

**Conclusions:**

Compared with conventional PCS, FCS for FNF fixation achieves comparable union rates while showing potential advantages in reducing postoperative complications such as femoral neck shortening and ONFH.

## Introduction

Hip fracture is a common type of fracture, with its global incidence projected to reach 6.26 million by 2050 [1], which represents a 1.9-fold increase from the 2018 level [2]. Each year, approximately 4.5 million people worldwide suffer from disabilities caused by hip fractures [3, 4]. Hip fractures primarily occur in older adults, typically as a result of osteoporosis and low-energy falls [5, 6], [7]. In contrast, such fractures in younger patients are usually due to high-energy trauma [8]. Femoral neck fractures (FNFs) account for approximately 50% of all hip fracture cases [9]. The treatment options vary depending on the patient’s age and injury mechanism. Elderly patients predominantly undergo arthroplasty, whereas younger patients typically rely on internal fixation to preserve hip joint function [10, 11]. Currently, arthroplasty has become a well-established procedure with favorable postoperative recovery outcomes; however, the efficacy of internal fixation still fails to achieve satisfactory results [12, 13]. According to the literature, the incidence of postoperative complications can be as high as 40%, and the probability of ONFH is 20% [14, 15]. The occurrence of complications significantly impacts patients’ postoperative quality of life and carries the risk of requiring secondary hip arthroplasty. Consequently, there is an urgent need to develop optimal internal fixation techniques to minimize postoperative complications. Cannulated screw fixation, as one of the predominant internal fixation modalities, has gained global acceptance due to its technical advantages including easier operability and reduced blood loss [16]. However, this technique is still associated with a relatively high incidence of postoperative complications [17]. Recent studies have proposed utilizing fully-threaded cannulated screw (FCS) as an alternative to partially-threaded screw (PCS) for FNF fixation [18, 19]. Compared to conventional PCS, the fully threaded design provides superior biomechanical stability, offering enhanced resistance to shear and shortening [20]. However, current clinical studies on FCS remain limited in both quantity and sample size, and their fixation efficacy continues to be debated. Therefore, we conducted a retrospective study to evaluate the safety and efficacy of FCS in the internal fixation of FNFs, with the aim of providing evidence-based guidance for clinical practice.

## Materials and methods

### Study design

This study was a retrospective, single-center investigation conducted at a large tertiary trauma center from January 1, 2019 to December 31, 2023. This research followed the ethical guidelines specified in the 1964 Declaration of Helsinki. Approval of the study design was granted by the Medical Ethics Committee of the Hospital. According to the Institutional Review Board, informed consent was waived.

### Patients selection

**Fig. 1:**
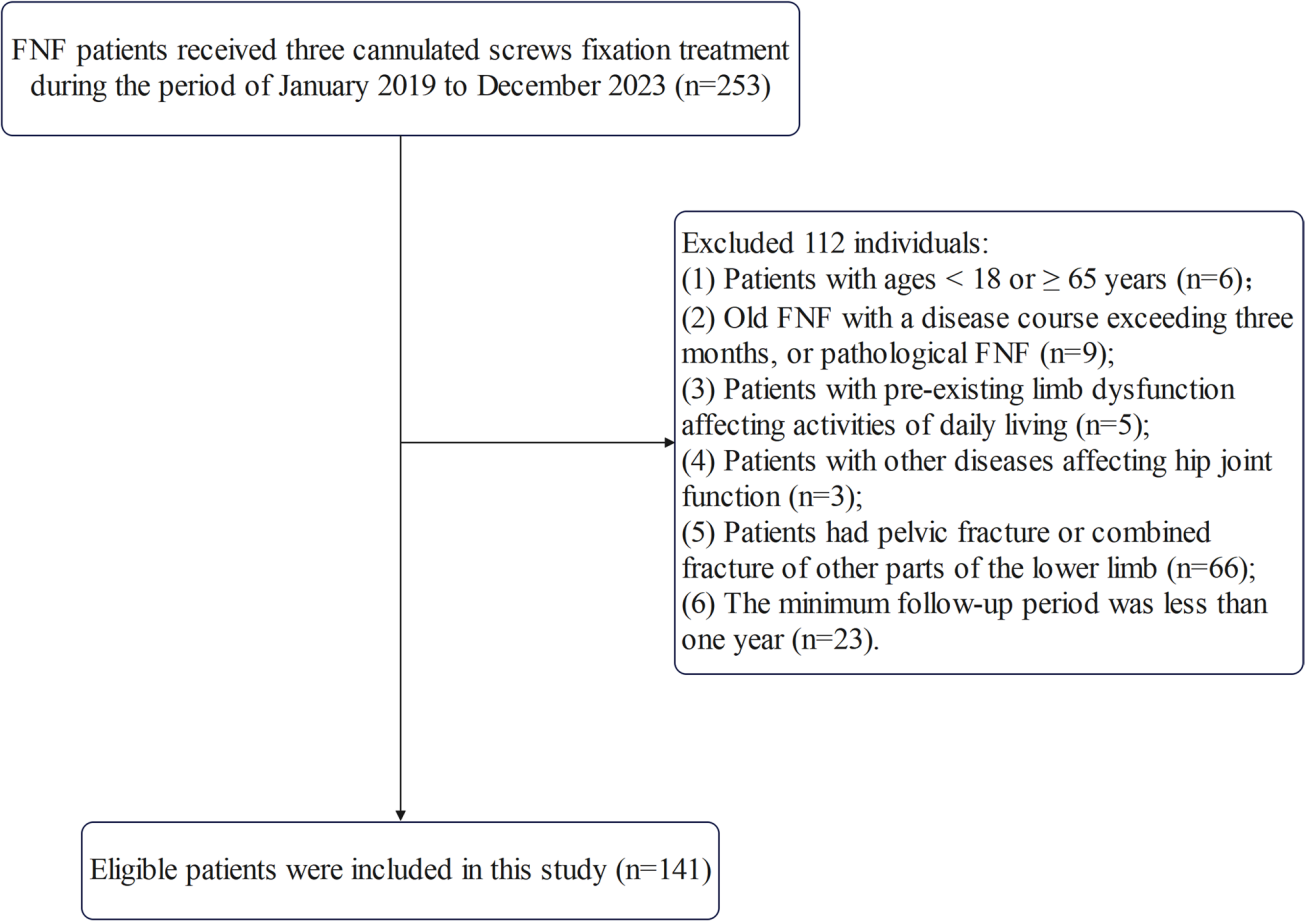
Patient selection process

The inclusion criteria were as follows: patients (1) Diagnosed with FNF based on medical history, physical examination, and imaging studies and (2) Treated with three cannulated screws, which were either PCS or headless, tapered, variable-pitch FCS. The exclusion criteria were as follows: (1) Patients with ages < 18 or ≥ 65 years; (2) Old FNF with a disease course exceeding 3 months, or pathological FNF; (3) Patients with pre-existing limb dysfunction affecting activities of daily living; (4) Patients with other diseases affecting hip joint function; (5) Patients had pelvic fracture or combined fracture of other parts of the lower limb and (6) The minimum follow-up period was less than 1 year. A total of 141 patients were ultimately enrolled in this study, with 80 cases allocated to the PCS group and 61 cases to the FCS group (Fig. 1).

### Surgery and follow-up

**Fig. 2:**
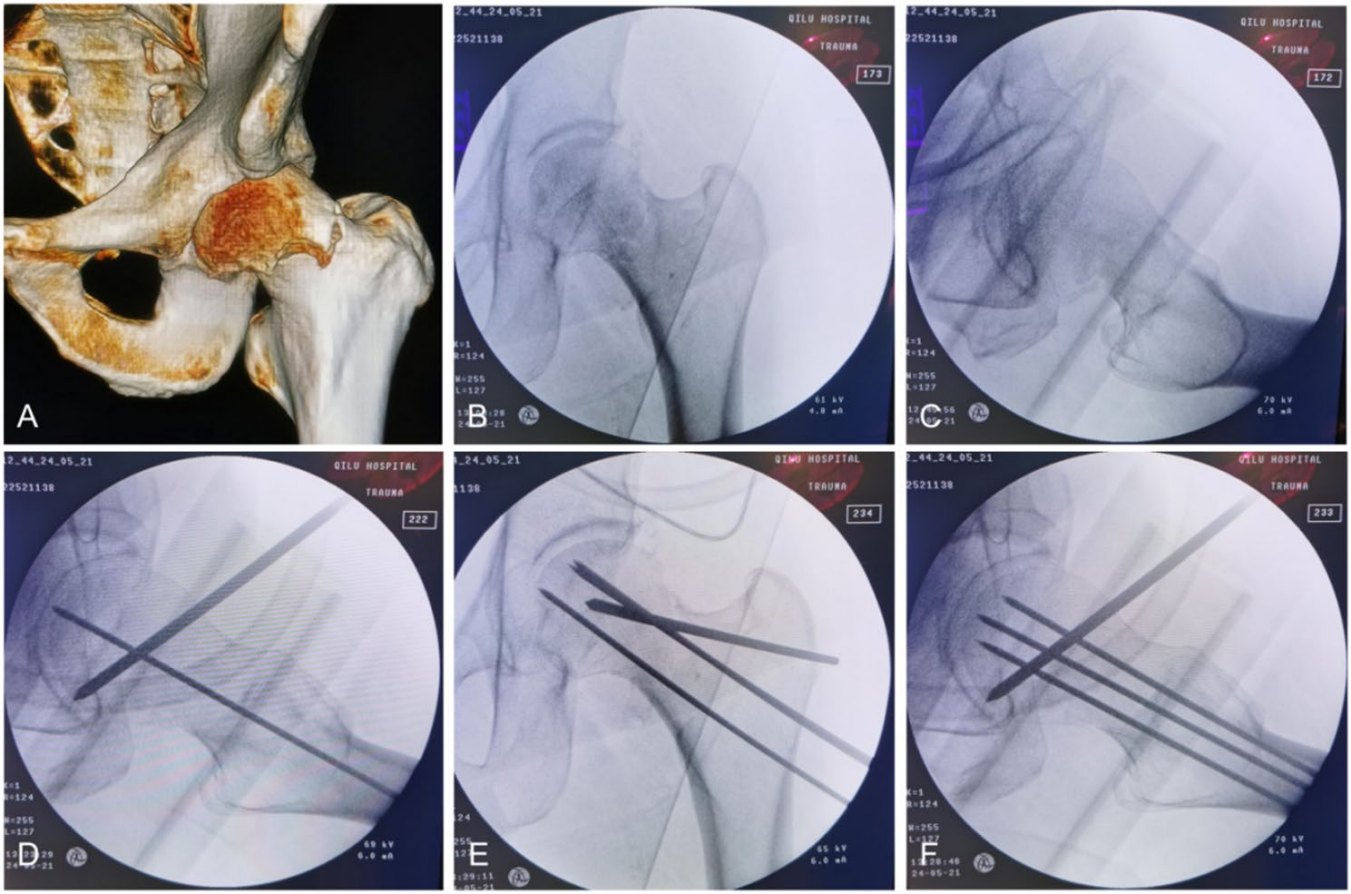
A 45-year-old male with a left femoral neck fracture (Garden III, Pauwels II). **A** Preoperative CT scan. **B**, **C** Despite traction, adduction, and internal rotation, significant posterior tilt of the femoral head persists. **D** Reduction was achieved by levering the femoral head anteriorly using a Steinmann pin and Kirschner wires. **E**, **F** Three guide pins were placed in an inverted triangular configuration

All patients were operated on by the same surgical team. If closed reduction fails to achieve satisfactory reduction quality, open reduction was adopted. Following reduction, the Kirschner wires were used for temporary fixation, and satisfactory fracture reduction was confirmed under fluoroscopy (Fig. 2). Three guide wires were placed in an inverted triangle parallel configuration to the femoral head through the femoral neck. After confirming optimal wires placement by fluoroscopy, depth was measured, and three cannulated screws were sequentially inserted along the guide wires. When fracture alignment is satisfactory with no gap between the fracture ends, the screws should be inserted in the following sequence: first the posterosuperior screw, followed by the inferior screw, and finally the anterosuperior screw. If a gap persists between the fracture ends, the sequence should be adjusted as follows: first the anterosuperior screw, then the inferior screw, and lastly the posterosuperior screw. Anteroposterior and lateral radiographs of the hip were reviewed within three days after surgery. Reexamination was performed at 6 weeks, 3 months, 6 months, and 1 year after surgery, and rehabilitation training was performed according to fracture healing.

### Outcome measurements

**Fig. 3:**
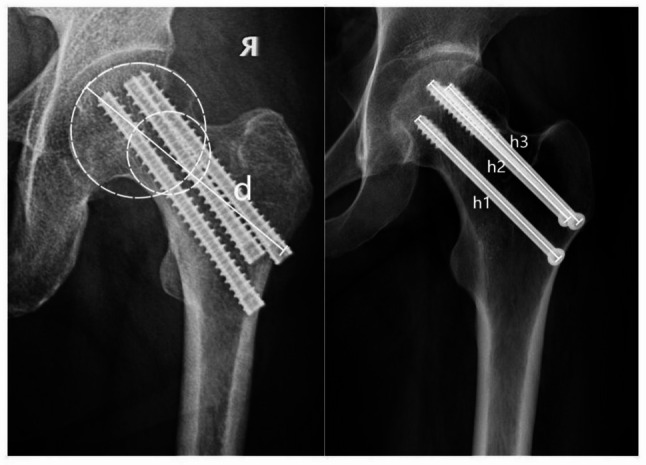
Measuring method of femoral neck shortening length

The influencing factors included: gender, age, body mass index (BMI), comorbidities, type of internal fixation, fracture classification and Garden’s alignment index. Fracture classification includes two aspects: Pauwels classification [21] and Garden classification [22]. According to the Pauwels classification, fractures are classified as stable (Pauwels I and II) and unstable (Pauwels III); according to the Garden classification, fractures are classified as nondisplaced (Garden I and II) and displaced (Garden III and IV). Preoperative bone mineral density (BMD) was assessed using T-scores according to the WHO standards for osteoporosis [23], with a T-score less than − 2.5 indicating osteoporosis [24]. The Garden’s alignment index was assessed on immediate post-reduction intraoperative X-rays to evaluate the quality of reduction [25]. Garden Type I is defined as 160 degrees on the anteroposterior view and 180 degrees on the lateral view. Garden Type II is defined as between 155 and 180 degrees on both the anteroposterior and lateral views. Garden Type III is defined as either the anteroposterior or lateral view being less than 155 degrees or greater than 180 degrees. Garden Type IV is defined as both the anteroposterior and lateral views falling outside the range of 155 degrees to 180 degrees. The outcome measures included osteonecrosis of the femoral head (ONFH), nonunion, femoral neck shortening, and internal fixation failure. ONFH was assessed radiologically according to Ficat’s criteria [26]. The occurrence of femoral head necrosis was defined as the presence of any of the following findings on any radiograph taken between the postoperative period and the final follow-up: uneven density of the femoral head, cystic changes, osteosclerosis, the crescent sign, subchondral bone collapse, deformation of the femoral head, acetabular lesions, joint space narrowing, or varying degrees of osteoarthritic changes. Nonunion was defined as a fracture that failed to heal within 9 months of injury with 3 consecutive months of healing stagnation [27]. Femoral neck shortening was defined as ≥ 5 mm shortening of the femoral neck on the final follow-up radiograph compared with the first postoperative radiograph [28]. Fixation failure was defined as screw cut-out, implant breakage, varus collapse (< 120° neck-shaft angle), or severe fracture shortening (≥ 10 mm) [13]. Assessments of all outcome measures were completed independently by two orthopedic surgeons, and agreement was reached by discussion if disagreement existed. Femoral neck shortening was measured as shown in Fig. 3. The center of the femoral head is determined by the center of the best-fit circle. The axis of the femoral neck was determined by connecting the center of the femoral head to the center of the circle tangential to the cortex of the femoral neck. The initial length of the femoral neck axis was measured as d, while the postoperative measurement at 1-year follow-up was recorded as d′. Delta d = d − d′ is the change in femoral neck length. h is the screw length, ${\overline{\mathrm{h}}}$ = (h1 + h2 + h3)/3. h′ is the actual length of the screw. The length of the actual neck shortening Delta d′ = Delta d × h′ ÷ ${\overline{\mathrm{h}}}$. Femoral neck shortening was considered to occur if the femoral neck length was shortened by 5 mm or more.

## Statistical analysis

A chi-square test was performed for qualitative data such as gender, fracture type, comorbidities, and injured side in the two groups. Quantitative data such as age, BMI and surgical data were analyzed by normal analysis, and an independent sample t test was used if the data were normally distributed; otherwise, the Mann‒Whitney U test was performed. For the chi-square test, the Pearson chi-square test was used when the theoretical value T ≥ 5 and the total sample size n ≥ 40, and the adjusted chi-square test was used when 1 ≤ T < 5 and n ≥ 40; otherwise, Fisher’s exact test was used. Differences were considered significant when *p* values (2-sided) were < 0.05. We used SPSS version 27 (SPSS Inc., Chicago, IL, USA) for statistical analysis. Statistical significance was defined as having a *p* value less than 0.05.

## Results

### General characteristics

A total of 141 patients with FNFs met the inclusion criteria and were included in the analysis, including 80 patients in the PCS group and 61 patients in the FCS group. All patients presented with non-comminuted fractures. In the FCS group, there were 41 cases with one fully-threaded screw (placed posterosuperiorly), 14 cases with two fully-threaded screws (placed posterosuperiorly and anterosuperiorly), and 6 cases with three fully-threaded screws. Baseline data for both groups are presented in Table 1. There were no statistically significant differences between the two groups in gender, age, BMI, comorbidities, injured side or fracture type.

**Table 1:** General characteristics of patients in different internal fixation groups

Variables	PCS, n = 80	FCS, n = 61	*p* value
Gender (male) (n (%))	52 (65%)	35 (57.4%)	0.356
Age (years, median (IQR))	43 (18)	48 (17)	0.054
BMI, (kg/m^2^, median (IQR))	22.45 (4.39)	22.27 (3.71)	0.788
Comorbidities			
Osteoporosis (n (%))	8 (10%)	5 (8.2%)	0.716
Hypertension (n (%))	6 (7.5%)	4 (6.6%)	0.829
Diabetes mellitus (n (%))	5 (6.3%)	4 (6.6%)	0.943
Garden classification			
I–II (n (%))	37 (46.3%)	20 (32.8%)	0.107
III–IV (n (%))	43 (53.7%)	41 (67.2%)	
Pauwels classification			
I–II (n (%))	27 (33.8%)	13 (21.3%)	0.105
III (n (%))	53 (66.2%)	48 (78.7%)	
Injured side (left) (n (%))	43 (53.8%)	28 (45.9%)	0.355

### Primary outcomes

**Fig. 4:**
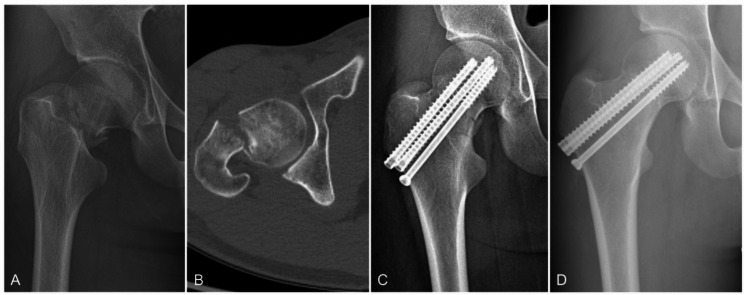
A 20-year-old male patient in the FCS group with a right FNF caused by falling (Garden type Ⅳ) (Pauwels type III). **A** Preoperative X-ray image. **B** Preoperative CT image. **C** Anteroposterior image at 2 days after the operation. **D** X-ray image at 6 months after the operation, showing continuous cortical and fracture healing without ONFH or internal fixation failure. The fixation was in a good position without significant withdrawal

**Fig. 5:**
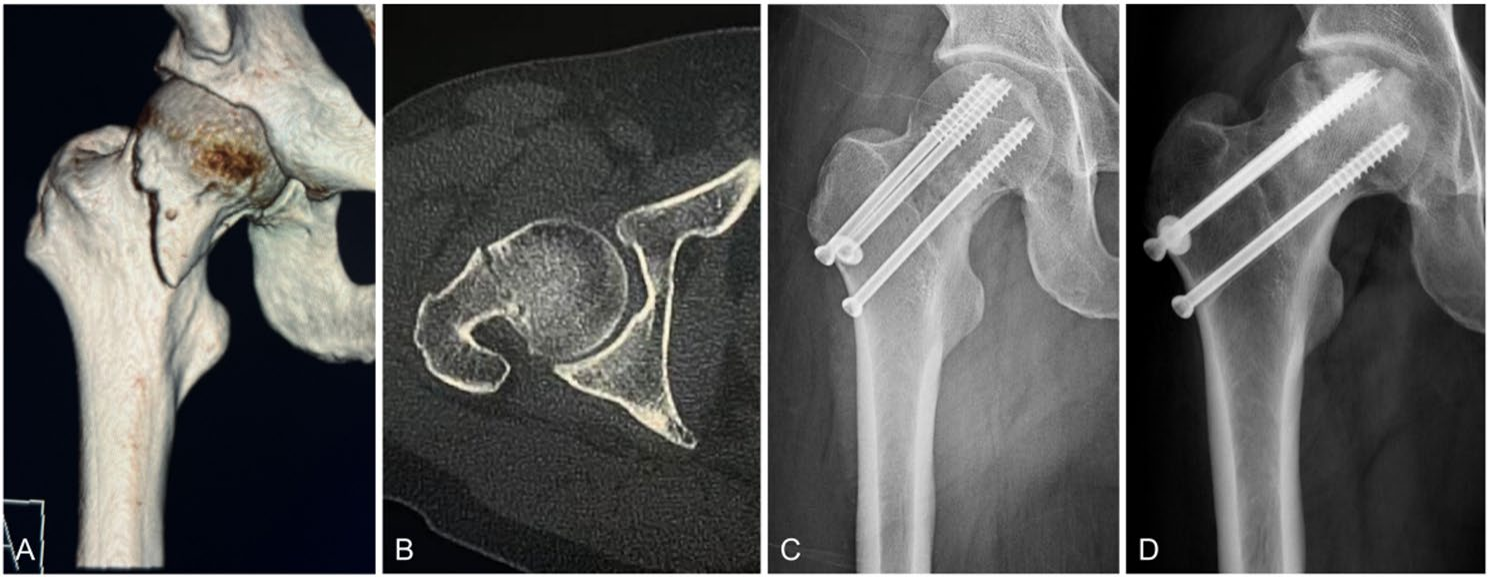
A 43-year-old male patient in the PCS group with a left FNF caused by falling (Garden type III) (Pauwels type III). **A** Preoperative three-dimensional reconstructed CT images, **B** Preoperative CT image. **C** X-ray image at 20 months after the operation, showing ONFH and femoral neck shortening

The incidence of postoperative complications in both groups is shown in Table 2. At the latest follow-up, ONFH was observed in 22 patients, with 17 cases (21.3%) in the PCS group and 5 cases (8.2%) in the FCS group, demonstrating a statistically significant difference (*p* = 0.034). Postoperative femoral neck shortening occurred in 25 patients, including 20 cases (25%) in the PCS group and 5 cases (8.2%) in the FCS group, which also showed statistical significance (*p* = 0.010). No significant differences were found between the two groups regarding nonunion rates or internal fixation failure rates. Furthermore, no surgical complications such as incision site infections, deep vein thrombosis, pulmonary embolism, or cardio-cerebrovascular events were reported (Figs. 4, 5).

**Table 2:** Prognoses of patients with different internal fixation groups

Variables	PCS, n = 80	FCS, n = 61	*p* value
ONFH rate (n (%))	17 (21.3%)	5 (8.2%)	0.034
Femoral neck shortening (n (%))	20 (25%)	5 (8.2%)	0.010
Nonunion rate (n (%))	5 (6.3%)	3 (4.9%)	0.735
Fixation failure (n (%))	3 (3.8%)	0 (0%)	0.126

### Secondary outcomes

According to the Garden’s alignment index criteria [25], the distribution of reduction quality was similar between the two groups. In the PCS group, 44 cases were graded as level I and 36 as level II, compared with 32 and 29 cases, respectively, in the FCS group, with no statistically significant difference in the index between groups. Additionally, no significant differences were observed in operative time, intraoperative blood loss, or fluoroscopy frequency between the two groups (Table 3).

**Table 3:** Secondary outcomes of two groups

Variables	PCS, n = 80	FCS, n = 61	*p* value
Garden’s alignment index			
Level I (n (%))	44 (55%)	32 (52.5%)	0.763
Level II (n (%))	36 (45%)	29 (47.5%)	
Surgical data			
Operative time (min, median (IQR))	85.01 ± 13.86	89.10 ± 17.68	0.116
Intraoperative blood loss (ml, mean ± SD)	42.31 ± 14.32	38.44 ± 11.78	0.077
Fluoroscopy frequency (mean ± SD)	14.36 ± 2.58	13.77 ± 2.74	0.190

### Subgroup analysis

Subgroup analysis were performed according to Garden classification (Table 4) and Pauwels classification (Table 5). Displaced fractures demonstrated significantly higher incidence rates of ONFH and femoral neck shortening compared with non-displaced fractures. Pauwels type III fractures showed markedly increased probabilities of developing ONFH and femoral neck shortening relative to Pauwels types I–II fractures.

**Table 4:** Comparison of displaced and non-displaced femoral neck fractures at the latest follow-up

Variables	Non-displaced, n = 57	Displaced, n = 84	*p* value
ONFH rate (n (%))	3 (5.3%)	19 (22.6%)	0.005
Femoral neck shortening (n (%))	3 (5.3%)	22 (26.2%)	0.001
Nonunion rate (n (%))	2 (3.5%)	6 (7.1%)	0.360
Fixation failure (n (%))	1 (1.8%)	2 (2.4%)	0.800

**Table 5:** Comparison of Pauwels I–II and Pauwels III femoral neck fractures at the latest follow-up

Variables	Pauwels I–II, n = 40	Pauwels III, n = 101	*p* value
ONFH rate (n (%))	2 (5.0%)	20 (19.8%)	0.029
Femoral neck shortening (n (%))	3 (7.5%)	22 (21.8%)	0.045
Nonunion rate (n (%))	1 (2.5%)	7 (6.9%)	0.305
Fixation failure (n (%))	1 (2.5%)	2 (2.0%)	0.847

To evaluate the potential heterogeneity within the FCS group due to differences in the number of screws, we performed a subgroup analysis. In the two-screw group, only one case of ONFH occurred postoperatively, while no complications were observed in the three-screw group (Table 6).

**Table 6:** Comparison of number of different fully-threaded cannulated screws

Variables	FCS group, n = 61			*p* value
	One, n = 41	Two, n = 14	Three, n = 6	
ONFH rate (n (%))	4 (9.8%)	1 (7.1%)	0 (0%)	1.000
Femoral neck shortening (n (%))	5 (12.2%)	0 (0%)	0 (0%)	0.448
Nonunion rate (n (%))	3 (7.3%)	0 (0%)	0 (0%)	0.585

## Discussion

In this retrospective study, we compared the clinical outcomes of FCS versus PCS for FNF fixation. Our analysis demonstrated that the full-thread design significantly reduced postoperative complication rates, with particular efficacy in preventing femoral neck shortening.

Given the limited lifespan of artificial hip prostheses, internal fixation remains the preferred treatment for FNFs in non-elderly patients [29]. Although various fixation options exist—including the femoral neck system (FNS), cannulated screws, and dynamic hip screws (DHS)—these techniques continue to be associated with high rates of postoperative complications such as ONFH and femoral neck shortening [30]. Cannulated screws have become the preferred treatment for most orthopedic surgeons due to their minimally invasive nature, limited blood loss, and operational convenience. The PCS represents a traditional fixation method for FNFs, whose design promotes fracture-end contact through a sliding compression mechanism to accelerate fracture healing [31]. However, its anti-shear capacity may be constrained by the distal-only thread distribution. The FCS represents a novel internal fixation device recently introduced for FNFs. Its full-thread design significantly enhances shear resistance and provides superior fracture-end grip strength [20]. Biomechanical studies have confirmed that FCS demonstrates notable advantages over PCS in both compressive strength and maximum load to failure [32]. However, clinical outcomes remain controversial, with substantial discrepancies across studies regarding its efficacy and potential advantages compared with traditional triple parallel partially threaded cannulated screws. Okcu et al. [18] conducted a prospective randomized study involving 44 cases and found that PCS were associated with shorter healing times and lower complication rates compared to FCS. In the FCS group, there were 8 complications, including 4 cases of nonunion and 4 cases of delayed union. In contrast, the PCS group had only 2 complications (1 nonunion and 1 varus malunion). Notably, no cases of ONFH were observed in either group. In a separate study of non-displaced FNFs, the two groups exhibited comparable rates of femoral neck shortening, osteonecrosis, nonunion, and other complications [33]. A retrospective controlled cohort study demonstrated that FCS significantly reduced postoperative femoral neck shortening in FNF patients (across all Garden classifications) [34]. However, these studies were limited by small sample sizes, potentially introducing selection bias.

In this study, we treated 141 FNF patients using either FCS or PCS, evaluating complication rates at final follow-up. Focusing on the incidence of ONFH, femoral neck shortening, nonunion, and internal fixation failure. Subgroup analysis were performed based on fracture classification and the number of FCS used.

FNF is an intracapsular fracture, the environment in which the fracture is located hinders the progress of fracture healing mechanism, the presence of synovial fluid hinders the hematoma formation necessary for secondary bone healing, and the lack of periosteum leads to the inability to provide proliferation and osteoblasts. Therefore, FNFs rely on primary bone healing [35]. Primary healing depends on the stability of the construct. The PCS design features proximal threads with a smooth shaft distal to the threads. The aim of this design is to achieve sliding compression when implanted in parallel to promote fracture healing. However, when the continuous sliding compression caused by the load force due to the contraction of hip muscles and partial weight-bearing of the affected limb after surgery will cause the loss of femoral neck length, and finally cause the shortening of the femoral neck and screw withdrawal [36]. Femoral neck shortening may lead to abductor weakness and impaired gait, ultimately reducing the patient’s quality of life [37, 38]. Screw withdrawal can cause local irritation, pain, and may prevent lateral recumbency and normal walking [39]. In contrast, FCS nail body has a thread distribution throughout the length and has a stronger holding force on the fracture end, which can make the femoral neck in a static stable state and create good conditions for fracture healing, avoiding the occurrence of femoral neck shortening and screw withdrawal after surgery [40]. Our study also confirms this theory, femoral neck shortening was significantly lower in FCS compared with PCS.

ONFH represents the primary indication for revision hip arthroplasty, constituting one of the most clinically significant complications warranting particular attention. Published literature reports a 20.6% incidence of ONFH following internal fixation of displaced FNFs in patients under 60 years old [41]. Pauwels type III FNFs present significant stabilization challenges due to their pronounced vertical shear forces, consequently demonstrating substantially higher postoperative complication rates compared with Pauwels types I–II fractures [42]. The full-threaded design enhances bone contact area. The trailing threads improve purchase in the lateral femoral cortex, while the central threads increase cut-out resistance within the femoral neck. This configuration stabilizes the femoral neck, creating favorable conditions for fracture healing [43]. A biomechanical study demonstrated superior stability of FCS compared with PCS, with significantly greater compressive strength and maximum load to failure, particularly at a Pauwels angle of 70° [44]. In this study, FCS demonstrated significant efficacy in reducing the incidence of ONFH, consistent with the conclusions of two small randomized controlled trials [45, 46]. Furthermore, our subgroup analysis confirmed significantly higher rates of ONFH in both displaced FNFs and Pauwels type III fractures postoperatively, which deserves our attention.

Nonunion and internal fixation failure are also among the reasons for reoperation. It has been reported that FCS does not slide and compress, and when bone resorption occurs at the fracture end, it may lead to an increased fracture gap, thereby increasing the risk of nonunion and reoperation rate [47]. The low incidence of nonunion and internal fixation failure in this study is not sufficient to draw definitive conclusions. Future clinical studies with larger sample sizes are needed to further validate the difference between FCS and PCS in fracture healing.

Limitations of this study include: (1) The single-center retrospective design may introduce potential selection bias; (2) The approximately 1-year follow-up period might be insufficient for evaluating long-term complications and functional outcomes following surgery; and (3) Our study cohort was relatively young, this limits the generalizability of our findings to the elderly osteoporotic population.

In summary, our findings demonstrate that compared with conventional PCS, FCS for FNF fixation achieves comparable union rates while showing potential advantages in reducing postoperative complications such as femoral neck shortening and ONFH. Based on current evidence, FCS can be considered a safe and effective option for internal fixation of FNFs, although its long-term efficacy requires further validation through larger-scale, multicenter prospective studies.

## Data Availability

Raw data are available by contacting the corresponding author on reasonable request.
